# Assessment of perfluoroalkyl substances concentration levels in wild bat guano samples

**DOI:** 10.1038/s41598-023-49638-5

**Published:** 2023-12-19

**Authors:** Slawomir Gonkowski, Julia Martín, Annemarie Kortas, Irene Aparicio, Juan Luis Santos, Esteban Alonso, Przemysław Sobiech, Liliana Rytel

**Affiliations:** 1 Department of Clinical Physiology, Faculty of Veterinary Medicine, University of Warmia and Mazury, Street Oczapowskiego 14, 10-719 Olsztyn, Poland; 2 Departamento de Química Analítica, Universidad de Sevilla, C/Virgen de África, 7, 41011 Sevilla, Spain; 3 Department of Internal Diseases with Clinic, Faculty of Veterinary Medicine, University of Warmia and Mazury in Olsztyn, Ul. Oczapowskiego 14, 10-719 Olsztyn, Poland

**Keywords:** Ecology, Environmental sciences, Risk factors, Chemistry

## Abstract

Perfluoroalkyl substances (PFASs) are substances commonly used in the production of various everyday objects, including among others kitchen dishes, cosmetics, or clothes. They penetrate to the environment and living organisms causing disturbances in the functioning of many internal organs and systems. Due to environmental pollution, wildlife is also exposed to PFASs, but the knowledge about this issue is rather limited. The aim of this study was to evaluate the exposure of wild greater mouse-eared bats (*Myotis myotis*), living in Poland, to six selected PFASs: five perfluoroalkyl carboxylic acids (perfluorobutanoic acid—PFBuA, perfluoropentanoic acid—PFPeA, perfluorohexanoic acid—PFHxA, perfluoroheptanoic acid—PFHpA, perfluorooctanoic acid—PFOA) and perfluorooctane sulfonic acid (PFOS) through the analysis of guano samples with liquid chromatography with tandem mass spectrometry (LC–MS–MS) method. To our knowledge this is the first study concerning the PFASs levels in bats, as well as using guano samples to evaluate the exposure of wild mammals to these substances. A total of 40 guano samples were collected from 4 bats summer (nursery) colonies located in various parts of Poland. The presence of PFASs mentioned were detected in all colonies studied, and concentration levels of these substances were sampling dependent. The highest concentration levels were observed in the case of PFPeA [1.34 and 3060 ng/g dry weight (dw)] and PFHxA (8.30–314 ng/g dw). This study confirms the exposure of wild bats to PFASs.

## Introduction

PFASs are a large group of synthetic fluoroorganic substances consisting of about 4000 chemical compounds, characterized by fully or partially fluorinated carbon chain, which may be linear or branched^1–3^. A characteristic feature of these substances are strong covalent bonds between fluorine and carbon atoms. Thanks to these bonds, PFASs are characterized by high chemical and thermal stability, which makes these compounds readily used in the production of many everyday objects, such as, among others, electronic components, food containers and kitchen dishes, waterproof clothes, cosmetics, carpets and even dental floss^4^. PFASs penetrate to food and the environment, and the common use of these substances in various industrial branches makes them one of the most important environmental pollutants, that have been found to be present in drinking and surface water, soil, air, plants and food all over the world^4–7^. PFASs may also penetrate to living organisms through the gastrointestinal tract, lungs, skin and placental blood in the prenatal period^8,9^, and the presence of these substances has been observed in the blood, urine, cerebrospinal fluid, various tissues and hair^10–12^. In the industry, the most commonly used PFASs are perfluorooctanoic acid (PFOA) and perfluorooctane sulfonic acid (PFOS). These substances are also the most studied and commonly found in the natural environment and living organisms^1–3^. Other substances belonging to PFASs and relatively often used in various branches of the industry are perfluorobutanoic acid (PFBuA), perfluoropentanoic acid (PFPeA), perfluorohexanoic acid (PFHxA) and perfluoroheptanoic acid (PFHpA). These substances are lees studied, however, it is known that they may also pollute the environment and affect the living organisms^2,3^.

Previous studies have shown that PFASs have a significant negative impact on human and animal health, impairing the functions of many organs and systems. It is known that PFASs penetrate through the blood–brain barrier and affect the functions of the central nervous system, causing, among others, changes in synapses, disturbances in calcium homeostasis in neurons, neurobehavioral effects and cognitive disfunctions^13^. PFASs has also negative influence on reproductive system, lungs, kidneys, gastrointestinal tract, liver, immunological cells and many other internal organs^9,14–16^. Due to the multi-directional side effects of PFASs in the living organisms, the assessment of human and animal exposure to these substances is of great importance in contemporary toxicology. To evaluate the exposure to PFASs, various matrices are used including urine, blood serum, breast milk, hair, semen samples, and some other tissues^11,17–19^.

The presence of PFASs has been described in humans in various regions of the world, which indicates a significant risk of human exposure to these substances^9^. Similar situation has been noted in domestic animals, especially in dogs and cats, which live in the same conditions as humans and are exposed to similar environmental pollutants^11,20^. Contrary to humans, domestic animals and water wild animals, the knowledge about exposure of wild terrestrial mammals to PFASs is relatively scanty. However, it is known that such species, similarly to humans and domestic animals, are also highly exposed to PFASs, these substances can affect their health status and, as factors impairing the functions of the reproductive system and population size^21–23^. On the other hand, bats—a highly diversified group of vertebrates including over 1300 species- are very sensitive to any changes in the environment, including the content of toxic substances in it^24^. For this reason, bats are considered to be one of the best bioindicators of quality of the environment^24–26^. Anthropogenic changes and progressive environmental pollution lead to a continuous drastic decline in their population^26^. It should be pointed out that it is not known to what extend the bats are exposed to PFASs, if this group of substances have possible health impact on bats, and on the number on their population due to the lack of studies within this respective field.

Therefore the aim of this study was to determine the degree of exposure of greater mouse-eared bat (*Myotis myotis*), the most popular species of bat in Poland, to selected PFASs previously observed in the environment, including PFOA, PFOS, PFBuA, PFPeA, PFHxA, PFHpA through the guano samples analysis. To our knowledge, this study is the first evaluation of the degree of exposure of bats to PFASs and the first using guano samples to evaluate the exposure to PFASs in wild mammals.

## Results

PFASs were found in all guano samples included into the study. Cumulative results from all guano samples without considering the location of sample collection, are presented in Table 1, and individual concentration levels from particular samples are shown in Supplementary material (Table S3).

**Table 1:** Concentration levels (ng/g dw) and frequency of detection of PFASs in the analyzed guano samples (n = 40)—cumulative data.

Compound	Range	Arithmetic mean	Standard deviation	Median	Frequency of detection
PFBuA	< MQL-382	22.8	70.6	7.89	70
PFPeA	1.34–3060	140.4	475.7	59.1	100
PFHxA	8.30–314	48.6	47.1	46.3	100
PFHpA	< MQL-494	41.7	83.8	21.4	80
PFOA	4.31–44.4	7.09	6.1	5.94	100
PFOS	5.29–61.1	19.2	9.4	16.8	100

Compound acronyms: PFBuA, Perfluorobutanoic acid; PFPeA, Perfluoropentanoic acid; PFHxA, Perfluorohexanoic acid; PFHpA, Perfluoroheptanoic acid; PFOA, Perfluorooctanoic acid; PFOS, Perfluorooctanesulfonic acid. < MQL, Below Method Quantification Limit (PFBuA = 0.01 ng/g dw; PFHpA = 0.01 ng/g dw.)

PFPeA and PFHxA were the substances at the highest concentration levels, median concentrations amounted to 59.1 and 46.3 ng/g dry weight (dw), respectively. Both compounds were quantified on all samples measured. Slightly lower concentration levels were observed in the case of PFHpA (median value 21.4 ng/g dw) and PFOS (median value 16.8 ng/g dw), whereby the concentration level of PFOS higher than method quantification limit (MQL) was noted in 100% of samples, and PFHpA in 80%. PFBuA and PFOA were the compounds at the significantly lower concentration levels. Median concentration of PFBuA (observed in concentration above MQL in 70% of samples) amounted to 7.89 ng/g dw. PFOA median concentration achieved 5.94 ng/g dw (with frequency of detection 100%). Several PFASs were noted in each guano samples, and concentration differences between samples were very pronounced and concentration levels in some samples extremely differed from values noted in other samples (especially in sample no. 9 from bats colony number 1) (Table S1).

Moreover, during the present investigation some differences in PFASs concentration levels and frequency of detection were noted depending on where the samples were taken (Table 2). The mean concentration levels (± SD) of PFPeA in location 4 was 5.9 $\pm$ 8.2 ng/g dw and it was statistically lower than concentration levels measured from other locations, in which values ranged from 67.9 ± 21.8 ng/g dw in location 2 to 400.2 ± 935.7 ng/g dw in location 1. Between location 1, 2 and 3 there were no statistically significant differences in PFPeA concentration levels. Mean concentration levels of PFHxA reached up to 80.1 ± 83.0 ng/g dw, 43.1 ± 7.5 ng/g dw, 56.8 ± 8.6 ng/g dw and 14.1 ± 8.0 ng/g dw in locations 1, 2, 3, and 4, respectively. Statistically significant differences were noted between locations 1 and 4, as well as 3 and 4 (Table 2).

**Table 2:** Concentration levels (ng/g dw) and frequency of detection of PFASs in the analyzed guano samples taking into account the sampling place.

Bats colony number	1	2	3	4
PFPeA				
Minimum	42.4	50.4	56.7	1.3
Maximum	3060	123	122	28.7
Mean ± SD	400.2 ± 935.7^a^	67.9 ± 21.8^b^	87.5 ± 23.3^c^	5.9 ± 8.2^abc^
PFHxA				
Minimum	37.0	32.6	45.3	8.30
Maximum	314	55.1	74.2	32.1
Mean ± SD	80.1 ± 83.0^d^	43.1 ± 7.5	56.8 ± 8.6^e^	14.1 ± 8.0^de^
PFHpA				
Minimum	30.9	16.2	13.2	0.01
Maximum	494	34.3	31.4	8.30
Mean ± SD	91.1 ± 141.9^fgh^	20.8 ± 5.7^fi^	20.2 ± 4.8^gj^	1.3 ± 2.9^hij^
PFOS				
Minimum	11.0	15.70	22.60	5.290
Maximum	61.1	20.80	29.80	21.80
Mean ± SD	17.2 ± 15.5^k^	18.1 ± 1.7	27.8 ± 2.2^kl^	13.7 ± 4.1^l^
PFOA				
Minimum	5.050	5.190	5.470	4.310
Maximum	44.40	6.510	7.670	9.180
Mean ± SD	10.3 ± 12.0	5.8 ± 0.4	6.5 ± 0.8	5.8 ± 1.4
PFBuA				
Minimum	7.15	0.01	0.01	4.150
Maximum	382	9.70	11.2	9.140
Mean ± SD	51.0 ± 116.4^mn^	4.6 ± 3.6^m^	1.1 ± 3.6^n^	7.1 ± 1.5

Measurements below MQL were treated as 0.01. Statistically significant differences are marked based on Krusal-Wallis test were found for all parameters except for PFOA. a–n—pairwise significant differences based on Dunn test with Bonferroni adjustment.

The lowest concentration levels of PFHpA (1.3 ± 2.9 ng/g dw) were found in location 4. This value was significantly statistically lower than values noted in other locations, where concentration levels of PFHpA ranged from 20.2 ± 4.9 ng/g dw (location 3) to 91.1 ± 142.0 ng/g dw (location 1) (Table 2). Additionally, significant differences were confirmed between locations 1 and 2 as well as between locations 1 and 3.

PFOS concentration levels ranged from 13.7 ± 4.1 ng/g dw in location 4 to 27.8 ± 2.2 ng/g dw in location 3 (Table 2), and statistically significant differences were found between locations 1 and 3, as well as between locations 3 and 4. For PFBuA concentration levels ranged from 1.1 ± 3.6 ng/g dw in location 2 to 51.0 ± 116.4 ng/g dw in location 1 (Table 2). Significant statistical differences were found between locations 1 and 2 as well as 1 and 3.

In turn, PFOA concentration levels ranged from 5.8 ± 0.4 ng/g dw in location 2 to 10.3 ± 12.0 ng/g dw in location 1 (Table 2). Contrary to the other PFASs, there were no statistically significant differences in levels of PFOA between particular locations.

During correlation analysis it was found that PFBuA was positively correlated with PFHpA with medium strength (rho = 0.43) and negatively correlated with PFOS also with medium strength (rho = -0.52). PFPeA was correlated significantly with all other PFASs, except for PFBuA, with positive direction and moderate strength (PFOA and PFOS, rho = 0.49 and rho = 0.36, respectively) or high strength (PFHxA and PHpA, rho = 0.84 and rho = 0.81, respectively). PFHxA was positively correlated with PFPeA, PFHpA, PFOA and PFOS with moderate strength (PFOA and PFOS, rho = 0.54 and rho = 0.39, respectively) or high strength (PFPeA and PFHpA, rho = 0.84 and rho = 0.79, respectively). Positive correlations were identified between PFHpA and PFBuA, PFPeA, PFHxA and PFOA with moderate strength (PFBuA and PFOA, rho = 0.43 and rho = 0.49, respectively) or high strength (PFPeA and PFHxA, rho = 0.81 and rho = 0.79, respectively). PFOA was correlated with PFPeA, PFHxA and PFHpA with positive direction and moderate strength each (rho = 0.49, rho = 0.54 and rho = 0.49, respectively). PFOS was moderately correlated with negative direction with PFBuA (rho = 0.52) and moderately correlated with positive direction with PFPeA and PFHxA (rho = 0.36 and rho = 0.39, respectively) (Table 3).

**Table 3:** Outcomes of correlation analysis for PFASs in the analyzed guano samples (n = 40).

Compound	PFBuA	PFPeA	PFHxA	PFHpA	PFOA	PFOS
PFBuA	**1.00**	–	–	–	–	–
PFPeA	0.20	**1.00**	–	–	–	–
PFHxA	0.10	0.84*	**1.00**	–	–	–
PFHpA	0.43*	0.81*	0.79*	**1.00**	–	–
PFOA	-0.02	0.49*	0.54*	0.49*	**1.00**	–
PFOS	-0.52*	0.36*	0.39*	0.02	0.27	**1.00**

Table presents Spearman correlations coefficients. Measurements below MQL were treated as 0.01.

Significant values are in bold.

*Statistically significant (*p* < 0.05).

## Discussion

### Feces samples as a matrix study on exposure to PFASs

This study clearly showed that wild bats are exposed to PFASs. Such studies have not been carried out on this species of animals so far. On the other hand, previous observations on wild animals are in agreement with present study and have described the presence of PFASs in various matrices (mainly in blood and other tissues) of invertebrates^27^, fish^28,29^, birds^30^ and mammals^22^. However, It should be underlined that the assessment of exposure to PFASs in wild terrestrial mammals have been performed relatively rarely (Table 4). Moreover, the presents results, in which the majority of correlation between PFASs analyzed were positive, suggest that living organisms are exposed simultaneously to some substances from this group.

**Table 4:** Comparison of PFASs concentration levels (ng/g in solid matrices and ng/ml in liquid matrices) in wild terrestrial mammals detected by different authors worldwide.

Location	Species	n	Matrix	PFOA	PFOS	PFBuA	PFPeA	PFHxA	PFHpA	References
Austria	Chamois	11	Liver	< 0.1	< 0.1–4.6		< 1–2.3	< 1.0	< 1–4.3	Riebe et al.^40^
China	Siberian tiger	116	Blood	0.667–3.963	0.163–1.399				0.02–0.167	Wang et al.^23^
East Greenland	Polar bear	19	Brain	n.d-6.34	7.94–100					
Germany	Wild boar	91	Liver	< 0.3**–**114	< 0.3–1084					Kowalczyk et al.^22^
Germany	Wild boar	529	Liver	< 5.0–45	< 5–1780					Stahl et al.^34^
Germany		306	Muscles	< 1–7.4	< 1–28.6					
Germany	Wild boar	75	Liver	< 0.024–32	140–890	< 0.04–5.9	< 0.014–4.2	< 0.002–2.8	0.73–6.8	Felder et al.^41^
Germany		33	Muscles		< 0.042–21					
Germany	Fox	40	Liver	< 1.0–2	3.2–320		< 1.0	< 1.0–1.4	< 1.0	Riebe et al.^40^
Japan	Wild rats	10	Blood	0.123–6.57	1.97 -38.1					
Japan	Wild rats	216	Blood	0.05–60	0.05–148					
Japan	Racoon dog	2	Liver	3.5–6.1	19–33					
Poland	Beaver	6	Liver	0.51–0.87	< 0.126–8.45		n.d	n.d	n.d	
			Brain	0.5–1.59	< 0.126–0.86		n.d	n.d	n.d	
			Tail	0.7–1.06	< 0.126–1.34		n.d	n.d	n.d	
			Peritoneum	0.66–1	< 0.126- 4.64		n.d	n.d	n.d	
			Subcutaneous adipose	0.55–1.04	< 0.126–3.65		n.d	n.d	n.d	
Sweden	Mink	101	Liver	< 0.2–9.9	< 0.8–21,800					Persson et al.^35^

n.d., no detected.

Interestingly, till now feces samples have not been used in investigations of exposure to PFASs in wild mammals, although such samples seem to be appropriate for two reasons. Firstly, feces samples are relatively easy to collect without stressing the animals and without actually interfering with their welfare, which is of great importance in the case of protected species. The using other matrices, such as blood or other tissues is connected with the necessity of capturing the animal or its death. On the other hand, during feces samples collection particular attention should be paid to extrinsic contamination of samples. Secondly, it is known that PFASs substances are excreted from the body through the digestive tract. Experiments conducted on the rodents and cynomolgus monkeys have shown that in spite of the fact that urinary excretion is the primary route of PFOA and PFOS elimination from organisms, but feces may be also used to study on the degree of exposure to these substances^31,32^. Although the concentration levels of PFASs in the feces were lower compared to urine or blood and effect of administration of these substances is visible later than in mentioned above matrices, the content of PFASs in feces reflects the degree of exposure to these substances^32^. Previous studies on metabolism and excretion of PFASs with the present results strongly suggest that feces matrix may be used to evaluate the exposure in wild mammals to PFASs. In previous studies, feces samples were used to evaluate the exposure to PFASs in cats and dogs^20^.

### Supposed factors affecting PFASs concentration levels in the guano samples

During the present study differences in the PFASs concentration levels in guano samples between bats colonies have been observed. It agrees with previous observations on other matrices, which have described clear correlations between the PFASs concentration levels in wild animals and regions where these animals live (Table 4). Such situation is closely related to various environmental pollution and PFASs concentration levels on water, food, soil and air in different regions. Moreover, differences in PFASs concentration levels even in bats colonies located in close proximity to each other as well as in particular samples collected in the same colony strongly suggests that the degree of exposure to PFASs depends on the local factors and even animals living in one area are exposed to these substances to varying degrees. Moreover, the presents results, in which the majority of correlations between concentration levels particular PFASs analyzed in the same guano sample were positive, suggest that living organisms are exposed simultaneously to some substances from this group. This fact, together with synergistic activity of various PFASs increases the risk of adverse effects induced by these substances.

In the present study, one of the samples reached a higher concentration level than the rest for all PFASs (sample no. 9 from bats colony 1). At first glance it would seem that sample has been polluted during or after collection. However, all samples were collected and stored in the same conditions, therefore pollution of sample during preparation for analysis should be excluded. Therefore, this fact may be probably connected with any local factor acting on one or few animals in the colony. This factor, as well environmental factors contributing to the exposure of bats to PFASs, are difficult to determine. This is even more difficult as, in the case of wild animals, many different factors can influence the level of PFASs in the body. Such factors include among others seasonal temperature changes, diet changes, body condition, status health or period of reproduction^33^.

However, in previous studies have also described such extremely differences in PFASs concentration levels between particular individuals. For example, it has been shown that PFOS levels in the liver of wild boar in Germany fluctuated from below 5 to 1780 ng/mg^34^, and in the liver of mink in Sweden fluctuated from below 0.8 to even 21800 ng/g^35^.

### The comparison of present results with previous studies concerning PFASs in the living organisms

As the present study is the first description of PFASs concentration levels in bats as well as the first using guano samples to evaluate the exposure of wild animals exposure to these substances, the comparison of present results with previous observations is difficult. These difficulties are connected with the using completely different matrices in previous studies on wild mammals (Table 4). It should be remembered that the main route of excretion of PFASs from the body is urine, and studies on experimental animals have shown that the level of PFASs in the feces is lower than in the blood or urine^32^. In the light of these observations, the degree of exposure of bats observed in these studies should be considered as relatively high. It is also higher than the degree of human exposure to PFASs in Poland where concentration levels of PFOA in human blood serum fluctuated from 1.2 to 16 ng/mL, PFOS from 1.6 to 116 ng/mL, PFHpA from 0 to 0.79 ng/mL and PFHxA from 0 to 2.4 ng/mL^36–39^. Moreover, concentration values of PFASs noted in the present study are higher than those observed in the feces of dogs and cats living in the USA, in which the total mean concentration levels of 13 substances belonging to PFASs amounted to 85.4 ± 94.5 ng/g and 54.7 ± 26.9 ng/g, respectively^20^.

Interestingly, previous studies have reported that PFOA and PFOS (as the substances most often used in industry) are the main PFASs, to which humans and animals are exposed, and levels of these two compounds noted in various matrices in humans and wild animals have been higher than levels of other PFASs^37,38,40,41^. Contrary to previous investigations, results obtained in the present study have shown that in bats guano collected from all colonies included into the study the concentration levels of PFBuA, PFPeA, PFHxA, PFHpA were higher than PFOA and PFOS. The reason of such situation is unknown, but probably it is connected with environmental factors influencing on the bats colonies studied and sources of PFASs.

In the light of previous studies, it is known that the main sources of exposure of living organisms to PFASs are connected with polluted drinking water and food^9,42^. Air and house dust pollution is also of great importance^43^. In the case of bats colonies relatively important roles in exposure of animals to PFASs may be connected with elements of attics, where colonies live, such as roof sealing foils, roofing membranes, mineral wool for thermal isolation and even foils laid out in the place where the bat colonies live, especially to protect the structure of buildings from the effects of bats' guano. This thesis is supported by previous studies which have described the presence of PFASs in various building materials, including among others mounting and sealing foam, façade material, polystyrene oriented strand board (OSB) wood and lacquer^44,45^.

### Limitations of the study

The limitations to the present study should also be mentioned. The first of them is the fact that with this method of sampling, it is not possible to determine the exposure of individual animals, but only of the entire bats colony. It is possible that some animals may be more at risk of PFASs than others, and these differences will not always be found. The second limitation is a relatively small number of samples included in the study. However, it should be remembered that guano of several individuals could be in one sample, which makes the work more cross-sectional. Moreover, this work does not answer how exposure to PFASs affects the health status and welfare of wild-living bats.

## Materials and methods

### Chemical reagents

All reagents were analytical grade unless otherwise specified. Chemical reagents used in the study: acetic acid, formic acid, ammonium acetate—from Panreac (Barcelona, Spain), HPLC-grade methanol and water—from Romil (Barcelona, Spain), PFBuA (98%), PFPeA (97%), PFHxA (≥ 97%), PFHpA (99%), PFOA (96%) and PFOS (≥ 98%) from Sigma-Aldrich (Steinheim, Germany), internal standard (IS) perfluorooctanoic acid-13C4 (PFOA-13C4) (99%) from Cambridge Isotope Laboratories (MA, USA). Individual stock standard solutions were prepared at 1000 mg/L in MeOH. Working solutions were prepared by dilution of the stock standard solutions in methanol.

### Guano samples collection

**Figure 1:**
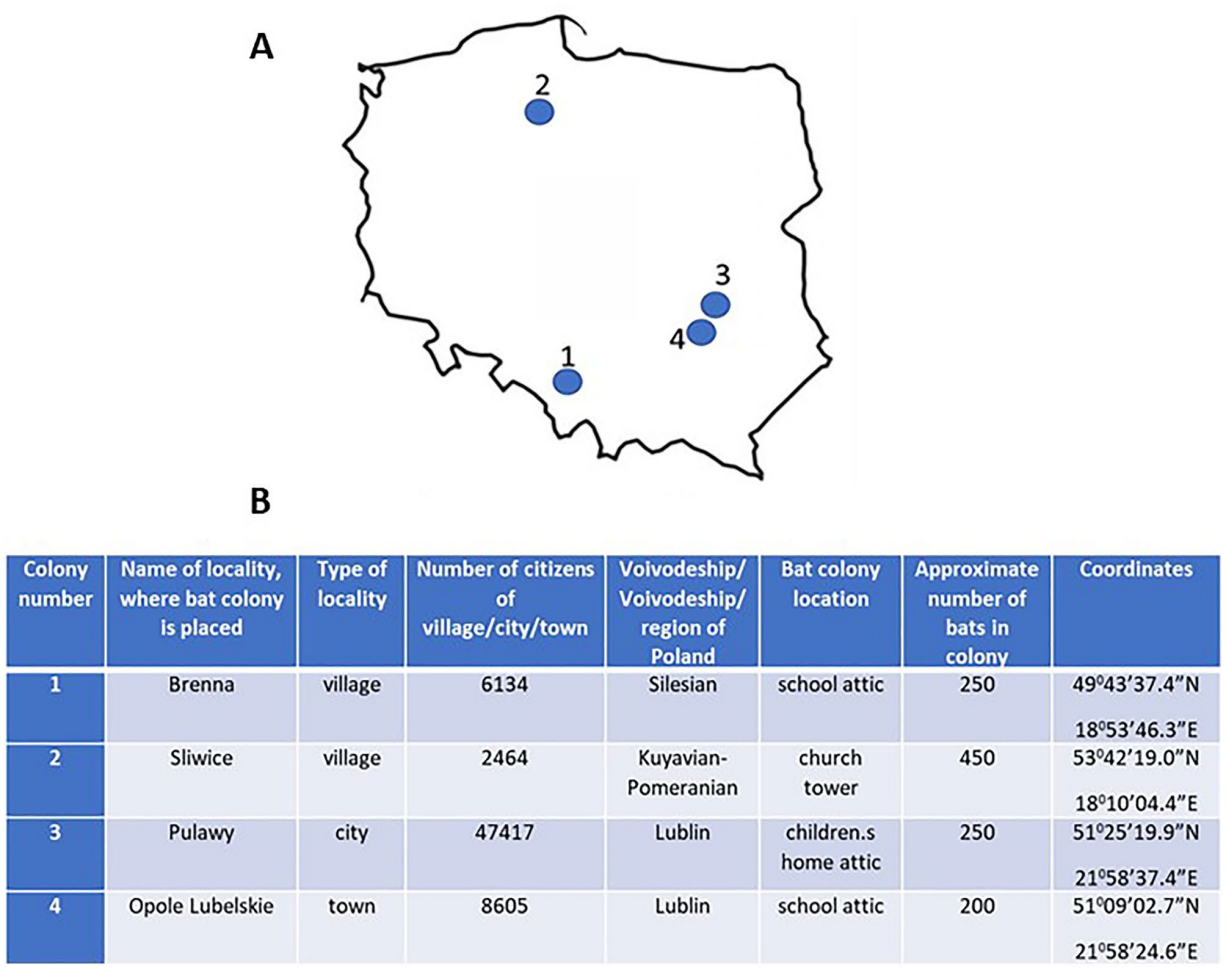
Location of bat colonies included into the study on the map of Poland (**A**) and their characterization (**B**).

Guano samples were collected in 4 bat summer (nursery) colonies of greater mouse-eared bats (*Myotis myotis*) located in various parts of Poland. The following colonies were included in the study (Fig. 1): (1) place: Brenna (number of citizens in 2016: 6134), colony location: attic of the school, coordinates: 49° 43′ 37.4″ N 18° 53′ 46.3″ E; approximate size of the colony: 250 bats; (2) place: Śliwice (number of citizens in 2011: 2464), colony location: church tower, coordinates: 53° 42′ 19.0″ N 18° 10′ 04.4″ E; approximate size of the colony:450 bats; (3) place: Pulawy (number of citizens in 2019: 47 417); colony location: attic of the children’s home, coordinates: 51° 25′ 19.9″ N 21° 58′ 37.4″ E, approximate size of the colony: 250 bats; (4) place: Opole Lubelskie (number of citizens in 2019: 8605), colony location: attic of the school, coordinates: 51° 09′ 02.7″ N 21° 58′ 24.6″ E; approximate size of the colony: 200 bats.

The selection of the colonies was not accidental and aimed to examine the content of PFASs in various distant regions of Poland. In turn, colonies 3 and 4 are relatively close to each other. Such selection was made to check whether the PFASs levels were not influenced by local factors.

Guano samples collection was performed in August and September 2021. For sampling, glass litter boxes were laid in different parts of the floor of the rooms where there were colonies of bats. After 48 h, the cuvettes were removed and the guano from the cuvettes was put into glass containers, frozen and stored at − 20 °C until further analysis. A total of 40 guano samples (10 from each colony of bats) were included in the research. During sampling particular emphasis was placed on avoiding disturbing and stressing the bats. Collection of guano samples was made up according to Act for the Protection of Animals for Scientific or Educational Purposes of 15 January 2015 (Official Gazette 2015, No. 266), applicable in the Republic of Poland. Because guano samples collection was a non-invasive procedure, which was not associated with stressing and scaring the bats and did not affect their welfare, consent was not required, approval from the Bioethical Committee for the present study was not required.

### Determination of PFASs in bat guano samples

#### Sample treatment

Guano samples were treated according the method described previously by Martin et al.^46^. Samples were freeze-dried, homogenized and grounded into powder. Lyophilized guano sample (1 g) was weighed into a 12 mL glass tube. Sample was fortified with 100 µL of a methanol solution of IS (250 ng/mL). The sample was sonicated with 7 mL of methanol (0.5% v/v, formic acid) as extraction solvent in a bath for 5 min and centrifuged for 5 min (4050 × g). The extraction procedure was repeated three times and the supernatants were combined.

To remove interferences from the matrix, a clean-up procedure, with 0.3 g of C18 sorbent was carried out. This step included shaking for 2 min and centrifugation for 5 min (4050 × g). Next, the solvent was evaporated to dryness under a nitrogen stream and the extract was reconstituted in 0.25 mL of a mixture methanol:water (50:50 v/v) and filtered through a 0.22 µm nylon filter. A 10 µL aliquot was injected into the liquid chromatography tandem mass spectrometry (LC–MS/MS) instrument.

#### Liquid chromatography–tandem mass spectrometry conditions

Chromatographic analyses were performed on an Agilent 1260 Infinity II (Agilent, Santa Clara, CA, USA). PFASs were analyzed using a LC method previously published by Martín et al.^46,47^. Separation was carried out using a HALO C18 Rapid Resolution (50 × 4.6 mm i.d., 2.7 µm). The mobile phase was composed of methanol (solvent A) and a buffer solution acetic acid/ammonium acetate (pH 4.4) (solvent B). The elution program was as follows: 0–14 min, linear gradient from 28 to 70% of solvent A, increased to 80% of A in 5 min and to 100% of A in 6 min and held for 2 min. Flow rate was 0.6 mL/min.

The LC system was coupled to a 6495 triple quadrupole mass spectrometer with electrospray ionization source operated in negative mode. Two multiple reaction monitoring (MRM) transitions, for identification and quantification purposes, were selected for each PFAS (Table S1).

### Validation requirements

The analytical features of the applied method are presented in Supplementary materials (Table S2). Due to the absence of certified material for PFASs in guano matrix, 6 g of spiked commercial bat guano were prepared containing the analytes at different concentration levels (0.01, 0.05, 0.25, 1.00, 5.00, 25.0, 50.0 and 100 ng/g dw for matrix matched calibration; and 5.00 and 50.0 ng/g dw for the quality control samples). Spiked samples were stirred and dried at room temperature until they recovered original weight. Then, spiked samples were ready for the sample treatment. Procedural blanks and quality control samples were injected by duplicate every 5 samples.

### Statistical analysis

PFASs concentration levels in total group were described with basic descriptive statistics (mean, standard deviation, median, minimum and maximum). Distribution normality was verified with Shapiro–Wilk test as well as skewness and kurtosis. No parameter had normal distribution in all four colonies, thus statistical analysis of the differences in PFASs concentration levels between particular bat colonies was performed with Kruskal–Wallis test. In case of significant outcome of Kruskal–Wallis test, Dunn test with Bonferroni adjustment was used to identify pairs of colonies with significant differences. Moreover, correlation analysis was performed to identify the direction and strength or relationships between analyzed PFASs. In all statistical calculations the differences were considered statistically significant at *p* < 0.05. The analysis was performed using GraphPad Prism version 9.2.0 (GraphPad Software, San Diego, CA USA) and R statistical software version 4.1.2.

## Conclusions

In conclusion, the present study in the first description of exposure of wild-living bats to PFASs, as well as the first using of guano samples to study the exposure of wild mammals to these substances. The obtained results clearly show that guano samples may be used in this type of studies, especially in cases, where minimal interference in the population of the animals is desired. The present study has shown that wild bats are exposed to PFASs to a relatively large extent, what suggests that bats may be the bioindicators of quality of the environment with regard to pollution with PFASs. It can be assumed that sources of PFASs acting on the bats are connected not only with polluted drinking water and/or food, but also with building materials of attics, where bats colonies live. Probably the exposure to PFASs impacts on the status health of bats, but proving this thesis requires further research. Particularly important issues for future research on this subject are questions how the concentration levels of PFASs in guano/feces samples correlate with the content of these substances in the blood and other organs and what PFASs concentration levels in guano/feces samples are dangerous for animals.

However, In spite of some limitations, the present investigation is the first step to further studies on factors affecting the exposure of wild bats to endocrine disruptors polluting the environment and their toxic impact on this mammal species.

### Supplementary Information

Supplementary Tables.

## Data Availability

All data that support the plot within this paper and other finding of this study are available from the corresponding author on reasonable request.
