# Supplementary material for: Assessment of perfluoroalkyl substances concentration levels in wild bat guano samples
Source: Sci Rep. 2023 Dec 19;13:22707. doi: 10.1038/s41598-023-49638-5 (PMC10733414; doi:10.1038/s41598-023-49638-5)
Supplement: Supplementary file 1 — Supplementary Tables. [file 41598_2023_49638_MOESM1_ESM.docx]

**Table S1.** MRM conditions used for LC-MS/MS of PFASs.

| **PFAS** | **Internal standard** | **MRM 1** | **MRM 2** | **Fragmentor**  **(V)** | **Collision energy (eV)** |
| --- | --- | --- | --- | --- | --- |
| **PFBuA** | PFOA-^13^C_4_ | 213> 169 | 213> 51 | 55 | 0 |
| **PFPeA** | PFOA-^13^C_4_ | 263 > 219 | 263 > 69 | 68 | 0 |
| **PFHxA** | PFOA-^13^C_4_ | 313 > 269 | 313 > 119 | 60 | 0 |
| **PFHpA** | PFOA-^13^C_4_ | 363 > 319 | 363 > 169 | 68 | 0 |
| **PFOA** | PFOA-^13^C_4_ | 413 > 369 | 413 > 169 | 68 | 4 |
| **PFOS** | PFOA-^13^C_4_ | 499 > 80 | 499 > 51 | 145 | 40 |

MRM 1: transition used for quantification; MRM 2: transition used for confirmation

**Table S2.** Linearity, method quantification limit, precision and recovery of PFASs in guano samples.

|  | **Linearity**  **R2** | **MQL (ng/g dm)** | **RSD**  **(%)** | **Rec**  **(%)** |
| --- | --- | --- | --- | --- |
| **PFBuA** | 0.999 | 0.01 | 6.0 | 90.0 |
| **PFPeA** | 0.996 | 0.01 | 5.4 | 85.9 |
| **PFHxA** | 0.997 | 0.01 | 4.5 | 88.1 |
| **PFHpA** | 0.999 | 0.01 | 9.6 | 86.8 |
| **PFOA** | 0.998 | 0.01 | 6.1 | 95.4 |
| **PFOS** | 0.996 | 0.01 | 6.9 | 91.2 |

MQL: Method quantification limit; RSD: Relative Standard Deviation; Rec: Recovery.

**Table S3.** Concentration levels (ng/g dw) of perfluoroalkyl substances in bat guano samples.

Compound acronyms: PFBuA: Perfluorobutanoic acid; PFPeA: Perfluoropentanoic acid; PFHxA: Perfluorohexanoic acid; PFHpA: Perfluoroheptanoic acid; PFOA: Perfluorooctanoic acid; PFOS: Perfluorooctanesulfonic acid. <MQL: Below Method Quantification Limit (PFBuA = 0.01 ng/g dw; PFHpA = 0.01 ng/g dw.)

| Bats colony | Sample | Concentration (ng/g) | | | | | |
| --- | --- | --- | --- | --- | --- | --- | --- |
|  |  | PFBuA | PFPeA | PFHxA | PFHpA | PFOA | PFOS |
| 1 | 1 | 11.0 | 91.3 | 49.9 | 41.0 | 7.23 | 11.1 |
|  | 2 | 11.0 | 45.6 | 42.8 | 35.0 | 5.87 | 11.8 |
|  | 3 | 7.83 | 51.9 | 61.8 | 43.1 | 6.94 | 11.1 |
|  | 4 | 7.15 | 42.4 | 37.0 | 30.9 | 5.17 | 11.0 |
|  | 5 | 17.7 | 170 | 55.2 | 50.1 | 7.36 | 13.1 |
|  | 6 | 22.2 | 143 | 54.6 | 45.7 | 5.05 | 12.9 |
|  | 7 | 18.1 | 145 | 70.4 | 58.3 | 6.75 | 13.0 |
|  | 8 | 18.5 | 129 | 42.5 | 47.3 | 6.15 | 13.2 |
|  | 9 | 382 | 3060 | 314 | 494 | 44.4 | 61.1 |
|  | 10 | 14.3 | 124 | 73.0 | 65.5 | 7.86 | 14.0 |
| 2 | 1 | 5.38 | 74.1 | 47.3 | 26.5 | 6.51 | 20.7 |
|  | 2 | 9.70 | 123 | 55.1 | 34.3 | 5.92 | 20.8 |
|  | 3 | <MQL | 64.2 | 32.6 | 16.2 | 6.15 | 17.8 |
|  | 4 | 8.21 | 50.4 | 40.0 | 20.5 | 5.98 | 17.9 |
|  | 5 | 4.17 | 80.2 | 48.6 | 22.7 | 5.59 | 18.1 |
|  | 6 | 7.59 | 58.8 | 40.3 | 17.0 | 5.19 | 17.3 |
|  | 7 | 5.63 | 56.8 | 53.1 | 17.2 | 5.23 | 16.2 |
|  | 8 | 5.26 | 68.7 | 41.3 | 17.6 | 5.67 | 15.7 |
|  | 9 | <MQL | 50.4 | 35.2 | 19.7 | 5.94 | 18.8 |
|  | 10 | <MQL | 52.7 | 37.9 | 16.5 | 5.54 | 17.8 |
| 3 | 1 | 11.2 | 122 | 75.2 | 31.4 | 5.76 | 27.3 |
|  | 2 | <MQL | 76.9 | 57.8 | 19.6 | 6.04 | 22.6 |
|  | 3 | <MQL | 58.5 | 45.3 | 16.6 | 5.47 | 25.9 |
|  | 4 | <MQL | 103 | 52.8 | 17.5 | 5.94 | 29.6 |
|  | 5 | <MQL | 89.6 | 57.0 | 17.9 | 6.05 | 28.3 |
|  | 6 | <MQL | 97.9 | 51.9 | 19.8 | 7.08 | 29.8 |
|  | 7 | <MQL | 59.4 | 56.0 | 22.0 | 7.49 | 29.7 |
|  | 8 | <MQL | 103 | 53.1 | 22.9 | 7.56 | 29.3 |
|  | 9 | <MQL | 108 | 69.0 | 20.9 | 7.67 | 27.4 |
|  | 10 | <MQL | 56.7 | 51.1 | 13.2 | 5.92 | 28.1 |
| 4 | 1 | 6.53 | 28.7 | 32.1 | 8.30 | 9.18 | 21.8 |
|  | 2 | 8.96 | 2.55 | 11.9 | <MQL | 6.44 | 11.6 |
|  | 3 | 7.59 | 6.38 | 8.62 | <MQL | 4.31 | 14.1 |
|  | 4 | 9.14 | 3.32 | 12.2 | <MQL | 5.59 | 14.0 |
|  | 5 | 4.15 | 6.20 | 25.3 | 4.60 | 6.69 | 5.29 |
|  | 6 | 6.92 | 2.75 | 11.2 | <MQL | 5.21 | 13.5 |
|  | 7 | 7.05 | 1.84 | 8.30 | <MQL | 5.08 | 13.2 |
|  | 8 | 5.70 | 1.34 | 12.8 | <MQL | 5.21 | 12.1 |
|  | 9 | 7.18 | 3.31 | 10.0 | <MQL | 5.70 | 15.7 |
|  | 10 | 7.94 | 2.44 | 9.01 | <MQL | 4.58 | 15.8 |
